# Bcl-2 Promoter Sequence G-Quadruplex Interactions with Three Planar and Non-Planar Cationic Porphyrins: TMPyP4, TMPyP3, and TMPyP2

**DOI:** 10.1371/journal.pone.0072462

**Published:** 2013-08-20

**Authors:** Vu H. Le, Narayana Nagesh, Edwin A. Lewis

**Affiliations:** 1 Department of Chemistry, Mississippi State University, Mississippi State, Mississippi, United States of America; 2 Centre for Cellular and Molecular Biology, Hyderabad, Andhra Pradesh, India; University of Quebect at Trois-Rivieres, Canada

## Abstract

The interactions of three related cationic porphyrins, TMPyP4, TMPyP3 and TMPyP2, with a WT 39-mer Bcl-2 promoter sequence G-quadruplex were studied using Circular Dichroism, ESI mass spectrometry, Isothermal Titration Calorimetry, and Fluorescence spectroscopy. The planar cationic porphyrin TMPyP4 (5, 10, 15, 20-meso-tetra (N-methyl-4-pyridyl) porphine) is shown to bind to a WT Bcl-2 G-quadruplex via two different binding modes, an *end binding* mode and a weaker mode attributed to *intercalation*. The related non-planar ligands, TMPyP3 and TMPyP2, are shown to bind to the Bcl-2 G-quadruplex by a single mode. ESI mass spectrometry experiments confirmed that the saturation stoichiometry is 4:1 for the TMPyP4 complex and 2:1 for the TMPyP2 and TMPyP3 complexes. ITC experiments determined that the equilibrium constant for formation of the (TMPyP4)_1_/DNA complex (K_1_ = 3.7 × 10^6^) is approximately two orders of magnitude greater than the equilibrium constant for the formation of the (TMPyP2)_1_/DNA complex, (K_1_ = 7.0 × 10^4^). Porphyrin fluorescence is consistent with intercalation in the case of the (TMPyP4)_3_/DNA and (TMPyP4)_4_/DNA complexes. The non-planar shape of the TMPyP2 and TMPyP3 molecules results in both a reduced affinity for the *end binding* interaction and the elimination of the *intercalation* binding mode.

## Introduction

The promoter region of the B-cell lymphoma-2 (Bcl-2) gene is guanine and cytosine rich [1–3]. Bcl-2 expression at low levels is required for cell survival; however, upregulation of the Bcl-2 gene can lead to formation of many common cancers and has been reported to play a role in resistance to conventional cancer treatments [2–4]. A 39 base pair region, located from 19 to 58 base pairs upstream of the Bcl-2 P1 promoter, plays a crucial role in the regulation of Bcl-2 transcription [5]. Dai et al used NMR and CD methods to demonstrate that the Bcl-2 39-mer purine rich strand folds into multiple intramolecular G-quadruplex structures [6].

G-quadruplex structures are thought to be generally involved gene regulation, with G-rich sequences found upstream from as many as 40% of all human genes [7]. Small molecules, which specifically interact with quadruplex DNA, have been shown to act as selective inhibitors of telomerase thereby demonstrating some potential as anti-cancer therapeutics [8–11]. Cationic porphyrins are known to associate with G-quadruplex DNA. Proposed binding modes include: groove binding (with or without self-stacking along the DNA surface), *end- stacking*, and *intercalation* [12–15]. The exact nature of the interactions between cationic porphyrins and G-quadruplex DNA depends on the folding topology and base sequence of the G-quadruplex and on the molecular structure of the porphyrin [16].

Haq et al. demonstrated that the saturation stoichiometry for porphyrin binding to G-quadruplex DNA depends on the number, n, of stacked G-tetrads and the formula (n+1) [13]. The Lewis group reported that the binding stoichiometry of the cationic porphyrin (5, 10, 15, 20-meso-tetra (N-methyl- 4-pyridyl) porphyrin), TMPyP4, to the c-MYC and Bcl-2 promoter region G-quadruplexes is 4:1 at saturation [17–19]. Their microcalorimetric and spectroscopic results were consistent with two TMPyP4 binding modes for both the c-MYC and Bcl-2 promoter quadruplexes; external or *end binding* and *intercalation* [17–19]. Wei et al. has similarly suggested that TMPyP4 binds to G-quadruplex DNA by a combination of binding modes that include external (end) stacking within the loop region and intercalation between G-tetrads with complex binding ratios of both 2:1 and 4:1 between TMPyP4 and G-quadruplex DNA [20]. In addition, Kumar et al. has reported that the concentration of porphyrin increases the relative concentration of quadruplex DNA in equilibrium with duplex DNA in dilute solutions [21].

The majority of previous studies have focused on the planar cationic porphyrin TMPyP4, although, Han et al. has described the interactions between the related ligands (5, 10, 15, 20-meso-tetra (N-methyl- 2-pyridyl) porphyrin), TMPyP2, and (5, 10, 15, 20-meso-tetra (N-methyl- 3-pyridyl) porphyrin), TMPyP3, with G-quadruplex forming oligonucleotides using gel mobility shift and helicase assays [22]. The structures of the three cationic porphyrins differ only in the location of the bulky N^+^–CH_3_ substituent group in the pyridinium rings. Steric hindrances force the four substituent pyridinium rings in the TMPyP2 and TMPyP3 molecules to be out of plane relative to the porphyrin ring. In the present study, we looked at the interaction of three cationic porphyrins (TMPyP2, TMPyP3, and TMPyP4) with a WT 39-mer G-quadruplex forming sequence from the Bcl-2 promoter region. Two of these ligands are non-planar (TMPyP2 and TMPyP3) and would be too bulky to thread between the stacked G-tetrads of the Bcl-2 promoter sequence G-quadruplex. Absence of a weaker “intercalation” binding mode for these non-planar ligands would serve to support our hypothesis that TMPyP4 binds to the Bcl-2 G-quadruplex (and other G-quadruplexes) by both end stacking and intercalation. The results of this study provide new insight into the origin of porphyrin/G-quadruplex DNA interactions, including the influence of ligand geometry and the influence of the intramolecular DNA folding topology on the thermodynamics for these interactions.

## Materials and Methods

The WT 39-mer Bcl-2 oligonucleotide used in this study was obtained from Oligos Etc (Wilsonville, OR). The Bcl-2 G-quadruplex forming promoter sequence, 5’-AGGGGCGGG

CGCGGGAGGAA GGGGGCGGGAGCGGGGCTG -3’, includes six runs of three or more guanines. Bcl-2 stock solutions were prepared by dissolution of weighed amounts of lyophilized oligonucleotide into KBPES {20 mM K_2_HPO_4_/KH_2_PO_4_, 2 mM K_4_EDTA} buffer with a supporting electrolyte concentration of 130 mM [KCl] and a pH of 7.0 [17]. Approximately 1 mL of the oligonucleotide was exhaustively dialyzed (1000 molecular-weight cutoff membrane) with two changes of buffer solution (1 L, 24h each) at 4°C. The concentrations of all DNA solutions were verified using ultraviolet–visible spectrophotometry (UV–Vis). Molar extinction coefficients were determined for each of the four oligonucleotides using a nearest-neighbor method for single stranded DNA [17,23]. The extinction coefficient at 260 nm for the 39-mer WT sequence was 3.863×10^5^ M^-1^cm^-1^.

**Figure 1:**
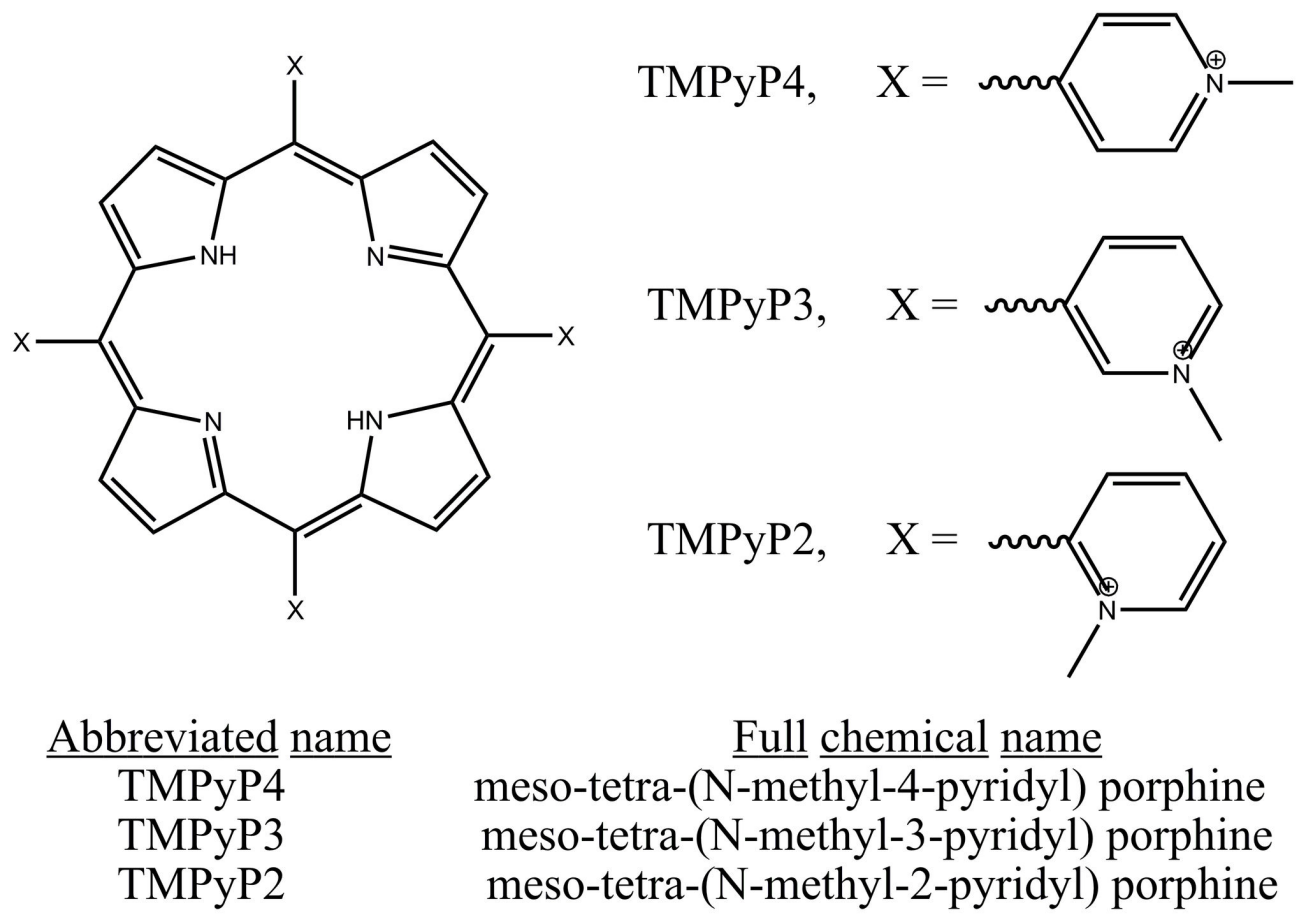
The chemical structures for the planar TMPyP4 and non-planar TMPyP3, and TMPyP2 porphyrin ligands.

TMPyP2, TMPyP3, and TMPyP4 were obtained from Frontier Scientific (Logan, UT). The chemical structures and naming nomenclature for these porphyrin ligands are given in Figure 1. All cationic porphyrin solutions were prepared by dissolution of a known amount of porphyrin into a measured volume of the oligonucleotide dialysate solution. This exact matching of the buffer composition in both the titrate (DNA) and titrant (porphyrin) solutions is necessary to minimize any buffer heat of dilution effects in the ITC experiments. The porphyrin solution concentrations were determined from UV–Vis absorbance measurements in the 414-424 nm region and the following molar extinction coefficients: TMPyP4, ε_424_=2.26 × 10^5^ M^-1^cm^-1^; TMPyP3, ε_417_=2.5× 10^5^ M^-1^cm^-1^; and TMPyP2, ε_414_=1.82 × 10^5^ M^-1^cm^-1^ [13].

CD analysis was performed using an Olis DSM 20 CD Spectrophotometer (Olis, Bogart, GA) equipped with a 1 cm path length quartz cuvette. All CD measurements and titrations were performed at 25^o^C in KBPES buffer (pH 7.0) with spectra collected over a wavelength range of 200-500 nm. The Bcl-2 promoter DNA concentration was nominally 5µM in G-quadruplex and a titration involved the addition of the porphyrin ligand to the DNA solution. CD spectra were collected at mole ratios (porphyrin: DNA) of 0: 1, 0.5:1, 1:1, 2:1, 3:1, 4:1, 5:1 and 6:1 for each of the three porphyrins and each of the four Bcl-2 oligonucleotides respectively.

ESI-MS experiments were performed using a Bruker MicrOTOFQ mass spectrometer in negative ion mode. Oligonucleotide/porphyrin samples were dissolved in a 50 mM ammonium acetate buffer (pH^≈^7) containing 20% methanol for these experiments. The WT-39-mer Bcl-2 was prepared at a concentration of approximately 80 µM in the ammonium acetate buffer and was exhaustively dialyzed at 4°C against the 50 mM ammonium acetate {NH_4_^+^/CH_3_CO_2_^-^} buffer. CD spectra for the oligonucleotide in 50 mM ammonium acetate were compared to CD spectra for the same samples in KBPES. The change to an ammonium acetate buffer did not affect the structure of the G-quadruplex, at least as determined by CD.

Stock solutions of the porphyrins were prepared by dissolution of the compound in the final dialysate to obtain a nominal porphyrin concentration of 1.0 mM. The ESI-MS samples were prepared by mixing the DNA and ligand stock solutions to prepare a mixture containing 4 equivalents of the porphyrin per equivalent of DNA. The MS capillary voltage was set to +4000 V, dry N_2_ gas flow was adjusted to 2.0 L/min at 110°C, and the G-quadruplex/porphyrin samples were directly introduced into the MS by using a kD Scientific syringe pump set to a flow rate of 108 µL/hour. Data processing was performed by using Bruker Daltonics Data Analysis program.

Isothermal Titration Calorimetry (ITC) experiments were performed using a Microcal VP-ITC (Microcal, Northampton, MA). All titrations were performed by over filling the ITC cell with ~1.5 mL of stock oligonucleotide solution having a concentration ranging from ~25–50µM. A titration involved the addition of approximately 55x5µL injections of the porphyrin titrant solution. TMPyP2, TMPyP3, and TMPyP4 titrant concentrations were nominally 1×10^-3^ to 3×10^-3^ M to obtain a ligand concentration approximately twenty times greater than oligonucleotide. All titrations were done at 25°C and at a supporting electrolyte concentration of 130 mM [K^+^]. Three replicate titration experiments were typically performed. The ITC data were fit using an independent sites model with either one or two independent sites required to fit the data within experimental error. The nonlinear regression fitting was done using CHASM data analysis program developed in our laboratory [24]. The ITC fitting procedures used here have been described previously [17,19,25–27].

Fluorescence emission spectra were collected using a FluoroMax-3 spectrofluorimeter (Horiba Jobin Yvon, Edison, NJ) equipped with a 1 cm path length quartz cuvette. The total concentration of the porphyrin was kept constant throughout the titration (900µM TMPyP2, or TMPyP3, and 1200 µM TMPyP4) while the concentration of the G-quadruplex DNA titrant increased from 0 to 300 µM over the course of the experiment. The TMPyP2, TMPyP3 and TMPyP4 solutions were excited at 424 nm, 417 nm and 433 nm, respectively. Emission spectra were collected from 600 to 800 nm after each injection of DNA into the porphyrin solution. All fluorescence measurements and titrations were performed at 25^o^C in KBPES buffer (pH 7.0).

## Results

**Figure 2:**
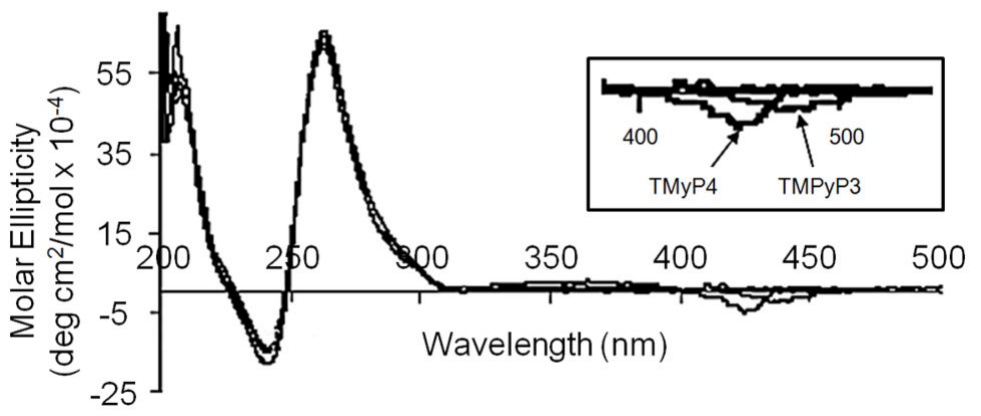
CD spectra for complex of the 39-mer Bcl-2 P1 promoter G-quadruplex saturated with bound TMPyP4, TMPyP3, or TMPyP2. The inset shows an expanded view of the CD spectra in the range of the porphyrin absorption spectrum. The very small negative CD signals in the 400-450 nm range are only observed in the presence of excess porphyrin for TMPyP4 and TMPyP3.

The interactions between the three cationic porphyrins (TMPyP2, TMPyP3, and TMPyP4) and the 39-mer WT Bcl-2 promoter sequence G-quadruplex motifs were probed using CD spectroscopy. The CD spectra for the 39-mer Bcl-2 promoter sequence quadruplex in the presence of saturating amounts TMPyP2, TMPyP3, and TMPyP4 are shown in Figure 2. The Bcl-2 39-mer porphyrin free spectrum is typical for a G-quadruplex structure with a predominantly parallel folding topology. Parallel G-quadruplex motifs typically exhibit a positive CD band near 264 nm and a negative CD band near 240 nm [1,6,28]. The small shoulder at 295 nm is an indication of minor species with anti-parallel folding topology in the 39Bcl-2 quadruplex mixture. The free cationic porphyrins do not possess a CD signal and porphyrin ligands bound to the G-quadruplex do not exhibit a significant induced CD signal. The obvious point to be made is that whether the porphyrin is self-stacking, stacked on the terminal G-tetrads in the quadruplex structure, or intercalated between G-tetrads in the quadruplex, the porphyrin environment in the complex is not asymmetric. The CD spectra for the 39-mer Bcl-2 promoter sequence quadruplex is unchanged by the addition of saturating amounts of TMPyP2, TMPyP3, or TMPyP4 either in CD band intensity or in CD band wavelength. The very low intensity negative bands in the 425 nm region (shown in the inset in Figure 2) are the likely result of very weakly bound porphyrin sitting in the quadruplex grooves. Induced CD signals are not seen in any of the TMPyP4 containing solutions, even at porphyrin/DNA ratios in excess of 4:1. The induced CD signals for the non-planar porphyrins only appear at ligand to DNA ratios that greatly exceed the saturation stoichiometry (2:1) for formation of these ligand DNA complexes. Our interpretation of these signals is that the G-quadruplex grooves could provide an asymmetric environment for binding excess porphyrin and that binding to these grooves might result in the observed CD spectrum. In the case of the planar ligand there are no induced CD signals indicating that neither end-stacking nor intercalation yield the required asymmetric environment for the porphyrin to exhibit an induced CD signal.

**Figure 3:**
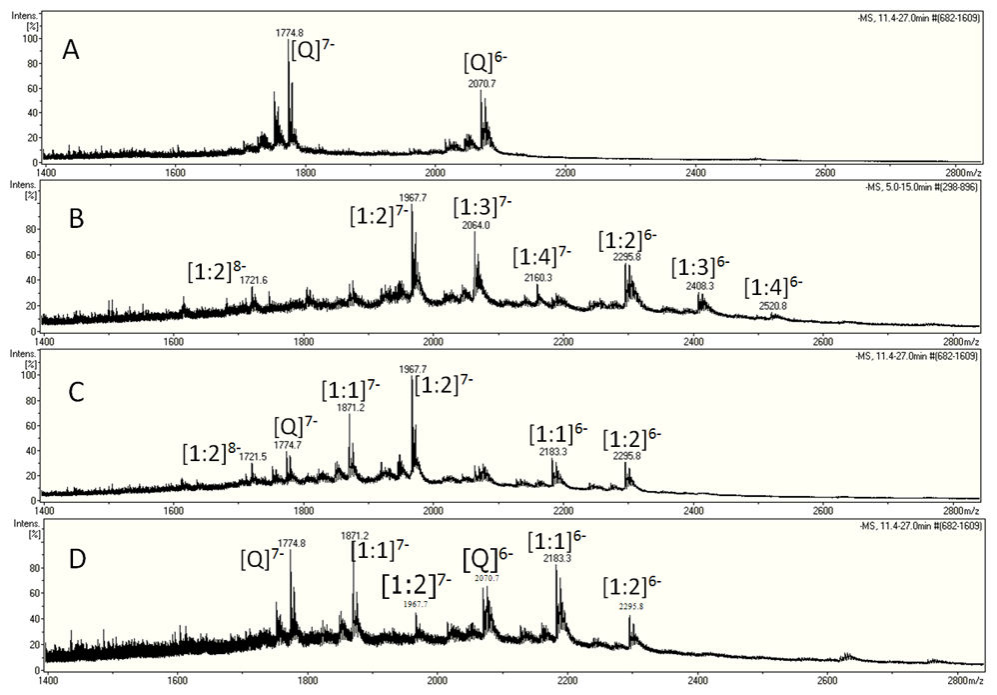
Electrospray ionization mass spectra for solutions containing (A) 80 µM WT 39-mer Bcl-2 G-Quadruplex and in complexation with (B) 320 µM TMPyP4 or (C) 320 µM TMPyP3 or (D) 320 µM TMPyP2.

ESI-mass spectrometry was used to determine the porphyrin/DNA species found in WT 39-mer Bcl-2 G-quadruplex solutions with and without saturating amounts of porphyrin ligands. The mass spectrum for free DNA is shown in Figure 3A, while the mass spectra for (TMPyP4)_x_/DNA, (TMPyP3)_x_/DNA and (TMPyP2)_x_/DNA are shown in Figure 3B, 3C, and 3D respectively. The mass spectrum for the TMPyP4/Bcl-2 (4:1) solution shows peaks corresponding to (TMPyP4)_x_/DNA complex species with x = 1, 2, 3, and 4, and having net charges of -6 to -8. There is no evidence of uncomplexed or free DNA in the TMPyP4 solution. The mass spectrum for the TMPyP3/Bcl-2 (4:1) solution shows peaks corresponding to uncomplexed DNA, and (TMPyP3)_x_/DNA complexes with x = 1 or 2 and having net charges of -6 to -8. In addition, there is an m/z peak at 1774.7 corresponding to some free DNA with a net charge of -7. The mass spectrum for the TMPyP2/Bcl-2 (4:1) solution shows peaks corresponding to uncomplexed DNA, and (TMPyP2)_x_/DNA complexes with x = 1 or 2 and having net charges of -6 and -7. The m/z peaks at 1774.7 and 2070.7 in the mass spectrum for the TMPyP2/Bcl-2 (4:1) solution correspond to free DNA with a net charge of -7 and -6 respectively.

**Figure 4:**
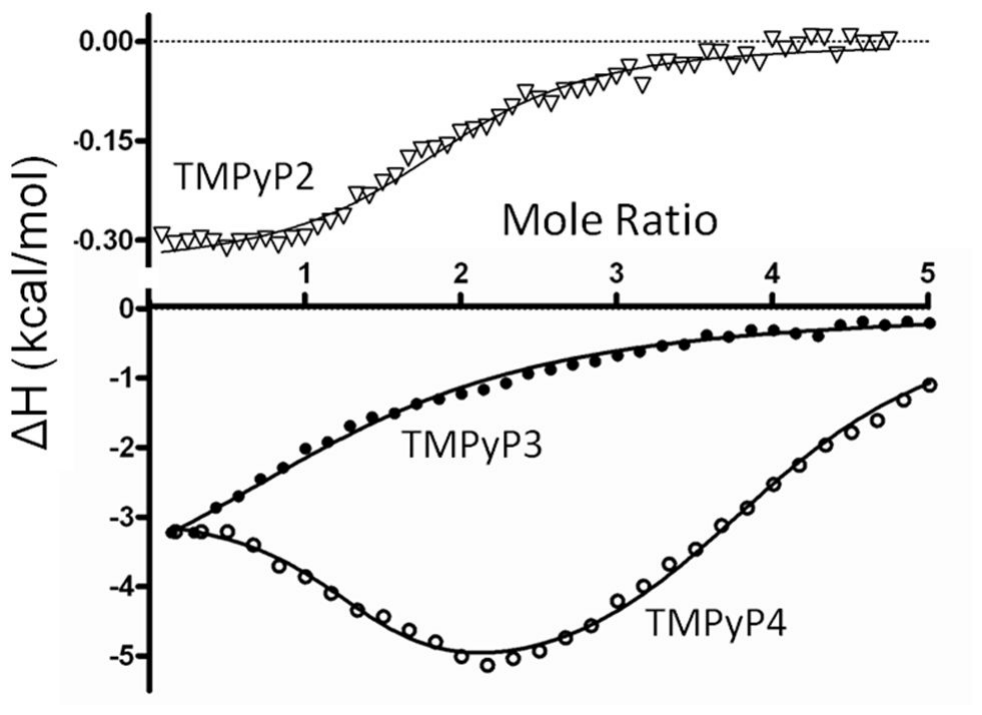
Typical ITC titration data (*points*) and nonlinear regression fits (*solid lines*) are shown for three titration experiments in which a dilute solution of porphyrin was added to a dilute solution of the annealed oligonucleotide in the calorimeter cell. The upper panel represents data for the titration of 39-mer Bcl-2 with TMPyP2 (Note the Y-axis is scaled from 0 to .3 kcals/mol). The lower panel represents data for the titration of 39-mer Bcl-2 with TMPyP3 and TMPyP4. The best fit parameters for the non linear regression analysis of these ITC data are given in Table 1.

Typical ITC data obtained for the titration of the 39Bcl-2 G-quadruplex DNA with TMPyP2, TMPyP3, or TMPyP4 at 25 ^O^C in 100 mM KBPES (pH 7.0) buffer are shown in Figure 4. The data points shown in each of the three representative thermograms are the corrected integrated heat produced per injection, plotted with respect to the mole ratio of porphyrin/DNA at each point in the titration. It needs to be noted that since the TMPyP2 affinity and enthalpy change are much smaller than for the other porphyrins, the TMPyP2 data in Figure 4 are shown on a magnified (10X) scale. After making corrections for blank heat effects and titrant dilution, the corrected heat data were fit to a thermodynamic model using a non-linear regression algorithm to obtain *best-fit* values for the equilibrium constant(s), *K*_i_, enthalpy change(s), ΔH_i_, and reaction stoichiometry, n, for complex formation. The TMPyP2 and TMPyP3 thermograms were fit with a model having a single binding mode, with two equivalent binding sites per DNA, and a saturation stoichiometry of 2:1. The TMPyP4 thermograms required a more complicated model having two binding modes, with each binding mode having two equivalent sites, and a saturation stoichiometry of 4:1. The *best-fit* values of the thermodynamic parameters estimated for the cationic porphyrin interactions with the WT 39-mer Bcl2 G-quadruplex DNA are listed in Table 1. Parameter values listed for the interactions of TMPyP4 with the WT 39-mer Bcl-2 promoter sequence G-quadruplex are very similar to the *K*_i_, ΔG_i_, ΔH_i_, and –TΔS_i_ values reported previously for the interaction of TMPyP4 with the shorter c-MYC and Bcl-2 promoter G-quadruplexes [17,19,29].

**Table 1:** ITC Derived Thermodynamic Parameters for the binding of porphyrin ligands (TMPyP2, TMPyP3, and TMPyP4) to the 39Bcl-2 promoter sequence G-Quadruplex.

**Ligand**	**K_1_ ×10^-5^**	**ΔG_1_ (kcal/mol)**	**ΔH_1_ (kcal/mol)**	**-TΔS_1_ (kcal/mol)**	**K_2_ ×10^-5^**	**ΔG_2_ (kcal/mol)**	**ΔH_2_ (kcal/mol)**	**-TΔS_2_ (kcal/mol)**
**TMPyP2**	0.7 ± 0.5	-6.6	-0.6 ± 0.2	-6.0	-	-	-	-
**TMPyP3**	2.6 ± 0.5	-7.4	-4.1 ± 0.1	-3.3	-	-	-	-
**TMPyP4**	37.0 ± 4.0	-9.0	-4.2 ± 0.1	-4.7	0.9 ± 0.5	-6.7	-9.8 ± 0.1	-3.1

**Figure 5:**
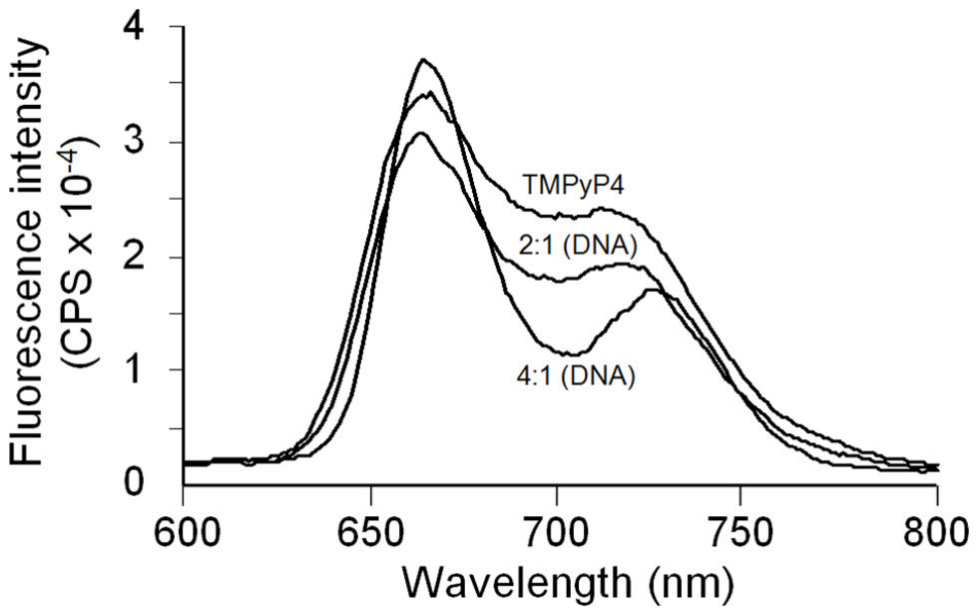
Fluorescence emission spectra obtained for the TMPyP4 in the presence or absence of 39-mer Bcl-2 G-quadruplex DNA. Spectrum 1 labeled TMPyP4 is for the free porphyrin (in the absence of DNA). Spectrum 2 is for TMPyP4 in the (TMPyP4)_2_ · 39-mer Bcl-2 complex. Spectrum 3 is for TMPyP4 in the (TMPyP4)_4_ · 39-mer Bcl-2 complex.

The interactions between the three cationic porphyrins and the WT 39-mer Bcl-2 promoter sequence G-quadruplex motif were also probed using fluorescence techniques. Emission spectra for the porphyrin ligands were recorded in DNA free solutions and in the presence of varying amounts of Bcl-2 promoter sequence quadruplex DNA. The fluorescence emission spectra observed for TMPyP4 in a G-quadruplex DNA free solution and in solutions containing either 2 moles of TMPyP4/mole of DNA or 4 moles of TMPyP4/mole of DNA are shown in Figure 5. In dilute solution, each of the three porphyrins exhibit complex emission spectra. TMPyP2 exhibits an emission spectrum with emission maxima at 640, 660, and 705 nm, while TMPyP3 exhibits emission maxima at 660 and 705 nm, and TMPyP4 exhibits emission maxima at 660 and in the range of 720 to 730 nm. In all instances, the total fluorescence emission is quenched in the presence of quadruplex DNA. This is a clear indication of porphyrin binding to G-quadruplex DNA. It is obvious, from the fluorescence spectra shown in Figure 5, that TMPyP4 fluorescence is changed on formation of the TMPyP4/39Bcl-2 complex. At low mole ratios of TMPyP4 to DNA (e.g. ratio ≤ 2.0), the TMPyP4 fluorescence is attenuated across the entire wavelength range of 625 to 775 nm. At higher mole ratios (e.g. TMPyP4/DNA ^≈^ 4:1), the TMPyP4 fluorescence emission spectrum changes dramatically, exhibiting a significant increase in fluorescence in the 660 nm region, a nonlinear attenuation near 700 nm, and a red shift in the λ_max_ for the 730 nm peak in the emission spectrum. Although in essence we are only showing two points in a DNA titration of TMPyP4 (Figure 5), the spectra shown represent the end points for formation of the 2:1 complex (with only end binding) and for the formation of the 4:1 complex with two different binding modes evident.

Changes in the emission spectra for TMPyP2 and TMPyP3 (data not shown) are linear with increasing DNA up to the point of saturation (2:1) porphyrin to DNA. The TMPyP2 and TMPyP3 fluorescence is quenched proportionally over the emission wavelength range (600 to 800 nm) by the DNA interaction. The results are consistent with bound TMPyP4 occupying two different environments in the saturated (4:1) TMPyP4/Bcl-2 promoter sequence quadruplexes while bound TMPyP2 and TMPyP3 appear to occupy a single environment in the saturated (2:1) complexes. The fluorescence experiments reported here for the interactions of all three porphyrin ligands with the WT 39-mer Bcl-2 promoter sequence agree in general with the CD, UV-Vis and ITC results reported previously for interaction of TMPyP4 with shorter c-MYC and Bcl-2 promoter sequence quadruplexes [17,19,29].

## Discussion

The WT 39-mer Bcl-2 oligonucleotide used in this study contains six contiguous runs having three or more consecutive guanines. The WT 39nt sequence has one run with five consecutive guanines, two runs containing four consecutive guanines, and three runs having three consecutive guanines. The Hurley group has reported that the WT 39-mer Bcl-2 P1 promoter sequence forms a mixture of three distinct intramolecular G-quadruplex structures in the presence of K^+^ ions [1,6]. Numerous others have shown that cationic porphyrins can bind to G-quadruplex DNA and stabilize the DNA structure through π-π stacking interactions between the aromatic porphyrin core and a G-tetrad or through electrostatic interactions between positively charged nitrogen atoms of the pyridyl rings and negatively charged phosphate oxygen atoms of DNA [1,13,14,22,30–32].

The CD results for the WT 39-mer Bcl-2 sequence used in this study agree with previously published data obtained for shorter Bcl-2 and c-MYC promoter sequence quadruplexes [17,19,29]. Specifically, each of the Bcl-2 promoter sequence G-quadruplexes (i.e. 23-mer, 27-mer, 30-mer and 39-mer) are best described as predominantly parallel structures exhibiting a large positive molar ellipticity in the 260 nm region with a smaller percentage of anti-parallel character (indicated by a small shoulder in the 295 nm region). In addition, the CD spectra for the Bcl-2 promoter quadruplexes is unchanged by the addition of the three porphyrin ligands up to saturation (2:1 for TMPyP2 or TMPyP3, and 4:1 for TMPyP4) and there is little or no evidence of an induced CD signal for the bound porphyrin (See Figure 2). It appears that bound porphyrin, either *end-stacked* or *intercalated*, has almost no effect on the CD spectra for the G-quadruplex structure or on the equilibrium between the ensemble of Bcl-2 G-quadruplex species.

The ESI-MS results provide spectroscopic evidence for the saturation stoichiometry of the ligand DNA binding. There is no detectable peak for free DNA in the TMPyP4/Bcl-2 mass spectrum. This is the obvious result of the large equilibrium constant for formation of the (TMPyP4)_x_/DNA complex species (K_1_^≈^ 4×10^6^ and K_2_^≈^ 7×10^4^). There are however, detectable peaks for free DNA in the mass spectra for both the (TMPyP3)_x_/Bcl-2 and (TMPyP2)_x_/Bcl-2 solutions. The TMPyP4/Bcl-2 spectrum (Figure 3B) clearly exhibits m/z peaks corresponding to TMPyP4/DNA species having 2:1, 3:1, and 4:1 complex stoichiometry. To the best of our knowledge, this is the first ESI-MS evidence for a G-quadruplex species having four porphyrin ligands bound to an intramolecular G-quadruplex. In the supplementary information, we provide ESI mass spectra obtained in similar experiments performed on a shorter 27-mer Bcl-2 G-Quadruplex construct (see Figure S1). The 39-mer Bcl-2 and 27-mer Bcl-2 ESI-MS results taken together suggest that the saturation stoichiometry of the G-quadruplex DNA/porphyrin ligand complexes is independent of the oligonucleotide length, eliminating the possibility of non-specific binding of porphyrin molecules to either the 5’ or the 3’ single stranded ends.

The ITC results present a consistent picture regarding the binding of the three porphyrin ligands to the WT 39-mer Bcl-2 promoter sequence quadruplex. We had previously reported on the site and mode of TMPyP4 interactions with the WT 27-mer Bcl-2 quadruplex and two mutant 27nt sequences designed to fold into 3:7:1 and 3:5:3 loop isomer quadruplexes [19]. In this previous work, we found that both mutant sequence quadruplexes exhibited lower affinity for TMPyP4 than the WT sequence and that the isomer with the largest (7 Base) unstructured loop showed an approximate 40-fold decrease in TMPyP4 binding affinity. The energetic profile reported here for the interaction of TMPyP4 with the WT 39-mer Bcl-2 sequence is similar to the energetic profiles reported previously for TMPyP4 binding to the WT Bcl-2 27mer, Bcl-2 (3:7:1) 23-mer loop isomer, and the Bcl-2 3:5:3 23mer loop isomer G-quadruplexes [19]. All of these quadruplex motifs exhibited two binding modes for TMPyP4 and a saturation stoichiometry of 4:1 in the ITC titration experiments. The higher affinity binding mode combines a favorable enthalpy contribution (-1.8 to -4.6 kcal/mol) with a more significant favorable entropy contribution of -4 to -9 kcal/mol in terms of (-TΔS). The lower affinity mode combines a more significant enthalpy contribution (-6 to -12 kcal/mol) with a smaller entropy term ranging from -3 to +5 kcal/mol. The energetic profiles reported here for the interactions of TMPyP3 and TMPyP2 exhibit only one binding mode that is most similar to the higher affinity TMPyP4 binding mode, at least in terms of the relative entropy and enthalpy contributions to the overall binding free energy change. Mode 1 binding affinity of the non-planar porphyrin molecules is reduced by a factor of 10 for TMPyP3 and more than 60 for TMPyP2 in comparison to TMPyP4 external binding or *end stacking*. Neither TMPyP3 nor TMPyP2 show Mode 2 binding, presumably the result of the non-planar molecules inability to *intercalate*.

We speculate here that the entropy-driven energetics for Mode 1 binding, typical for the binding of a hydrophobic ligand to the exterior of duplex DNA, can be used as a model for the exterior or end binding interactions of the TMPyP4, TMPyP3, and TMPyP2 ligands to the top or bottom of the G-quadruplex structure. The relative mode 1 affinities, TMPyP4 > TMPyP3 >> TMPyP2 are the result of the non-planar TMPyP3 and TMPyP2 molecules being unable to stack effectively on the flat or planar terminal G-tetrads. The energetic profile for the second TMPyP4 binding mode is best described as an enthalpy driven process. In the case of the insertion of planar aromatic ligands between the stacked bases in duplex DNA, the typical exothermic enthalpy change is largely the result of increased π-π stacking interactions between the DNA bases and the intercalated aromatic ligand [33–37]. We speculate here that the enthalpy driven energetics for Mode 2 binding, typical for the intercalation of a planar aromatic ligand into duplex DNA, can be used as a model for the stacking of the TMPyP4 ligand between two G-tetrads in the G-quadruplex structure. One subtle difference would be that in duplex intercalation there are additional consequences of intercalation, i.e. some unwinding of the DNA duplex.

Although the intercalation binding mode remains hypothetical, there continue to be reports that intercalation of planar ligands, like TMPyP4, can occur into G-quadruplex DNA structures [28,38–41]. Wei et al reported a change in the G-quadruplex DNA conformation on intercalation of TMPyP4 using spectroscopic methods [20]. Scheidt et al has shown by X-ray crystallography that the four 4-N-methyl pyridinium rings in a nitrosyl-Co II- TMPyP4 complex are nearly perpendicular to the plane of the core porphyrin ring system [42]. The interaction of TMPyP4 with G-quadruplex DNA in the (TMPyP4)_4_/DNA complex are maximized by rotation of the pyridinium rings to attain a planar TMPyP4 geometry favoring both end stacking on the terminal G-tetrads and intercalation between stacked G-tetrads in the G-quadruplex structure. The small change in G-quadruplex structure observed by Wei [20] can be attributed to the separation of two stacked G-tetrads required for intercalation to occur.

Gavathiotis et al reported an NMR structure (PDB 1NZM) having a spacing of 6.9 Å between a G- tetrad and a plane of four Adenines in a parallel stranded DNA quadruplex d(TTAGGGT)_4_ containing the human telomere repeat sequence and intercalated quinoacridinium ligands [38]. Keating and Szalai reported EPR data consistent with the intercalation of CuTMPyP4 into a d(T_4_G_8_T_4_)_4_ G-quadruplex [43]. Hounsou et al reported an NMR structure (PDB 2JWQ) having a spacing of 5.8 Å between a G- tetrad and a plane of four Adenines, with quinacridine based ligands (MMQs) intercalated between two G and A planes [39]. Cavallari et al have reported on an MD simulation study in which they found that TMPyP4 can stack with G-tetrads in the absence of interplane cations [41]. They report TMPyP4 G-tetrad stacking distances of 4.3-4.7 Å and that TMPyP4 intercalation depends on the length of the quadruplex, the stoichiometric ratio, and the edge termination motif. Although none of these previous reports directly demonstrates TMPyP4 intercalation between G-tetrads in an intramolecular G-quadruplex, we believe that intercalation of the more weakly bound porphyrin ligands remains a viable model for the (TMPyP4)_4_/DNA complex at this time.

The TMPyP2, TMPyP3, and TMPyP4 total fluorescence is attenuated on binding to the WT 39-mer Bcl-2 promoter sequence quadruplex. The attenuation of the porphyrin fluorescence on binding exhibits an irregular trend in that the percent fluorescence hypochromicity is largest for TMPyP3, followed by TMPyP4, and then TMPyP2. Porphyrin bound to the WT 39-mer Bcl-2 exhibits hypochromicity in total fluorescence of 53%, 29% and 13% for TMPyP3, TMPyP4, and TMPyP2 respectively at saturation (either 2:1 for TMPyP2 and TMPyP3 or 4:1 for TMPyP4). Whereas TMPyP2 and TMPyP3 exhibit attenuation of their emission spectra over the wavelength range of 600 to 800 nm when bound to quadruplex DNA, TMPyP4 fluorescence changes in a more complex manner on binding to the Bcl-2 quadruplexes. The decrease in fluorescence intensity for either TMPyP2 or TMPyP3 and for TMPyP4, at mole ratios of less than 2:1, is due to binding of the porphyrin externally or to the end of the G-tetrad core structure. The externally bound porphyrin remains exposed to solvent and its fluorescence quenched by water surrounding the externally bound ligand. The increase in the TMPyP4 fluorescence emission intensity, at mole ratios between 2:1 and 4:1 ligand/DNA, results from TMPyP4 intercalating between two G-tetrads resulting in energy transfer between the intercalated porphyrin and the eight guanines located in the two G-tetrads stacked on the TMPyP4. The same argument can be used to explain the apparent lower hypochromicity of bound TMPyP4 in comparison to bound TMPyP3 at low mole ratios (< 2:1). In effect, the increase in fluorescence emission for even a small amount of intercalated TMPyP4 (at low mole ratios) partially compensates for the attenuation of the externally bound TMPyP4 resulting in TMPyP4 exhibiting decreased hypochromicity in comparison to TMPyP3.

The results of the spectroscopic and microcalorimetric experiments described here clearly demonstrate that the planar cationic porphyrin, TMPyP4, binds with high affinity to the WT 39-mer Bcl-2 promoter sequence G-quadruplex by two distinctly different binding modes. We speculate that the two binding modes are an external or *end stacking* mode and an *intercalation* mode. The *intercalation* mode is eliminated when the bulky N^+^–CH_3_ pyridinium substituent groups (in TMPyP3 and TMPyP2) force the ligand to become non-planar, requiring a greater G-tetrad spacing for intercalation to occur. The fluorescence emission spectra can only be explained by the placement of the four bound TMPyP4 ligands in two completely different chemical environments in the saturated complex. The non-planar structure of the TMPyP2 and TMPyP3 ligands results not only in the loss of mode 2 binding but also in decreased affinity for the *end binding* interaction. The TMPyP2 ligand can only weakly stack on the terminal G-tetrads, while the TMPyP3 molecule can apparently partially rotate the pyridinium rings out of the way to gain a better π-π overlap between the aromatic porphyrin core and the terminal G-tetrads. The favorable binding interaction, that is inevitably lost in both the TMPyP2 and TMPyP3 quadruplex interactions, is any loop or terminal base sequence overlap with the end-stacked ligand.

The binding of the two non-planar porphyrins (TMPyP3 and TMPyP2) to the 39-mer Bcl-2 promoter sequence G-quadruplex is clearly different from the binding of the planar TMPyP4 ligand to the same DNA construct. The Structure Activity Relationships in these porphyrin binding interactions are the result of differences in the ligands solvent accessible surface area (SASA), charge location, and planarity. The weaker interactions for the non-planar ligands were anticipated in that all of these factors are working against the binding of the TMPyP2 and TMPyP3 molecules. Both TMPyP2 and TMPyP3 have somewhat smaller SASA values, both have reduced favorable charge/charge interactions as the positively charged pyridinium groups are moved further away from the negative charges in the DNA backbone in these complexes, and both have weaker π-π stacking interactions with the planar receptor G-tetrad. The non-planar ligands simply cannot stack as closely to an end tetrad or even fit between two stacked tetrads to intercalate. Even though these structure activity effects could have been predicted, their measurement removes any ambiguity relative to the placement of the bound porphyrins and is consistent with the two binding modes observed for TMPyP4, end-stacking and intercalation. The significance of this study is that it may be possible to design drug molecules that specifically target either the *end-stacking* site or the *intercalation* site in G-quadruplex motifs. If our binding model is correct, there are two unique opportunities for the future development of G-quadruplex structure-selective drugs.

## Supporting Information

Figure S1 **Electrospray ionization mass spectra for solutions containing (A) 80 µM WT 27-mer Bcl-2 G-Quadruplex and in complexation with (B) 320 µM TMPyP4 or (C) 320 µM TMPyP3 or (D) 320 µM TMPyP2.**
(TIF)
