# Supplementary figures and images for: Bcl-2 Promoter Sequence G-Quadruplex Interactions with Three Planar and Non-Planar Cationic Porphyrins: TMPyP4, TMPyP3, and TMPyP2
Source: PLoS One. 2013 Aug 20;8(8):e72462. doi: 10.1371/journal.pone.0072462 (PMC3748076; doi:10.1371/journal.pone.0072462)

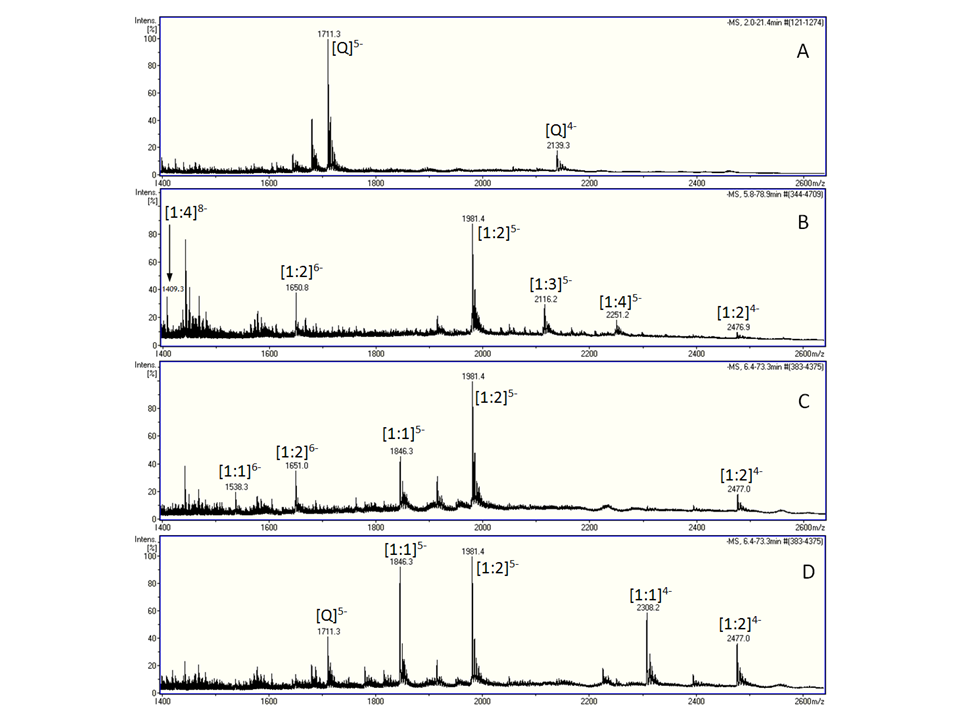

Supplement: Figure S1 — Electrospray ionization mass spectra for solutions containing (A) 80 µM WT 27-mer Bcl-2 G-Quadruplex and in complexation with (B) 320 µM TMPyP4 or (C) 320 µM TMPyP3 or (D) 320 µM TMPyP2. (TIF) [file pone.0072462.s001.tif]
